# All three mammalian MutL complexes are required for repeat expansion in a mouse cell model of the Fragile X-related disorders

**DOI:** 10.1371/journal.pgen.1008902

**Published:** 2020-06-26

**Authors:** Carson J. Miller, Geum-Yi Kim, Xiaonan Zhao, Karen Usdin

**Affiliations:** Section on Gene Structure and Disease, Laboratory of Cell and Molecular Biology, National Institute of Diabetes, Digestive and Kidney Diseases, National Institutes of Health, Bethesda, Maryland, United States of America; Duke University, UNITED STATES

## Abstract

Expansion of a CGG-repeat tract in the 5’ untranslated region of the *FMR1* gene causes the fragile X-related disorders (FXDs; aka the *FMR1* disorders). The expansion mechanism is likely shared by the 35+ other diseases resulting from expansion of a disease-specific microsatellite, but many steps in this process are unknown. We have shown previously that expansion is dependent upon functional mismatch repair proteins, including an absolute requirement for MutLγ, one of the three MutL heterodimeric complexes found in mammalian cells. We demonstrate here that both MutLα and MutLβ, the two other MutL complexes present in mammalian cells, are also required for most, if not all, expansions in a mouse embryonic stem cell model of the FXDs. A role for MutLα and MutLβ is consistent with human GWA studies implicating these complexes as modifiers of expansion risk in other Repeat Expansion Diseases. The requirement for all three complexes suggests a novel model in which these complexes co-operate to generate expansions. It also suggests that the PMS1 subunit of MutLβ may be a reasonable therapeutic target in those diseases in which somatic expansion is an important disease modifier.

## Author summary

Repeat Expansion Diseases, including the fragile X-related disorders, are a large group of human genetic disorders caused by a mutation in a disease-specific tandem repeat or microsatellite. This mutation increases the number of repeats in that microsatellite. Unusual features of this mutation include its high frequency and its absolute requirement for proteins involved in Mismatch Repair, some of the very proteins that normally protect against classical microsatellite instability. Proteins known to be essential for this mutation include MLH3, the MLH1 binding partner in MutLγ, one of the three mammalian MutL complexes. Here we show that PMS2 and PMS1, MLH1-binding partners in the remaining two MutL complexes, MutLα and MutLβ respectively, are also required for expansion in a cell-based model of the fragile X-related disorders. This has interesting implications for the mechanism of repeat expansion. It also identifies another potential therapeutic target for reducing the mutation responsible for these diseases.

## Introduction

The Repeat Expansion Diseases (REDs) are a large and seemingly ever-growing group of human genetic diseases arising from an increase, often a large one, in the number of repeats at a disease-specific microsatellite [1]. The fragile X-related disorders (FXDs; aka the *FMR1* disorders) are members of this group, arising as they do from expansion of a CGG-repeat tract in the 5’ untranslated region of the X-linked gene *FMR1* [2]. Many aspects of the expansion mechanism are still the subject of much debate (see [3, 4] for comprehensive reviews) and while there are likely to be significant similarities between the mutational mechanism that causes all of these REDs, the question of how many steps in the mutational pathway are shared is still unresolved.

We have previously shown that the set of DNA damage repair (DDR) proteins that affect repeat expansion in a knock-in mouse model of the FXDs overlap significantly with proteins implicated by Genome-Wide Association (GWA) studies as modifiers of somatic expansion, age at onset (AAO) and/or disease severity in humans with other REDs [5–11]. This includes FAN1, a nuclease best known for its role in the Fanconi Anemia pathway of DNA repair [12], as well as a number of proteins involved in mismatch repair (MMR) [13–18]. This suggests that the FXD mouse recapitulates important aspects of the REDs expansion mechanism.

Of all the DDR proteins implicated in causing expansion, those involved in MMR seem to be most critical for the process. For example, MutSβ, a heterodimer of MSH2 and MSH3, is one of the two lesion recognition complexes involved in MMR, and is important for expansions in most models of the REDs, including the FXDs [16, 19–23]. During typical MMR, the MutS complex binds to the mismatch and recruits MutL complexes to carry out later stages of MMR. Expansion in a number of models, including the FXD mouse, has been shown to also require MutLγ, one of the three MutL complexes seen in mammalian cells [13, 24, 25]. This finding was surprising since MutLγ is thought to be a minor player in MMR relative to MutLα, a MutL complex that is present in cells at much higher levels than MutLγ and whose loss causes much more microsatellite instability (MSI) [26–28].

Genome Wide Association Studies implicate both PMS2, the MLH1 binding partner in the MutLα complex, and PMS1, the MLH1 binding partner in the MutLβ complex, as modifiers of the AAO of REDs like Huntington Disease and many of the spinocerebellar ataxias [7, 9, 29]. However, conflicting results have been reported for the effect of MutLα in some model systems of different REDs. In the case of a mouse model of Myotonic Dystrophy type 1 (DM1), loss of PMS2 resulted in a ~50% decrease in the extent of somatic expansions [30]. In contrast, in a mouse model of Friedreich ataxia (FRDA), loss of PMS2 led to an increase in the expansion frequency in some tissues but not others [31]; while in a tissue culture model of this disease, shRNA knockdown of PMS2 using a lentiviral approach had no effect [24]. The basis of these very different effects on expansion is unclear. The contribution that PMS1 makes to expansion in these different models has, as yet, not been reported. The MutLβ complex is present at levels between those of MutLγ and MutLα [28]. PMS1 lacks the nuclease motif seen in PMS2 and MLH3 and, despite the abundance of MutLβ relative to MutLγ, its function remains elusive [32].

To evaluate the roles of the different MutL complexes in FXD repeat expansion in the FXDs, we have used a CRISPR-Cas9 approach to eliminate MLH3, PMS2 and PMS1 in a mouse embryonic stem cell (mESC) model of these disorders [33]. We demonstrate that all three MLH1 binding partners are required for expansion in this model system. This has implications both for the expansion mechanism and for potential therapeutic approaches to reducing somatic instability.

## Results

**Fig 1:**
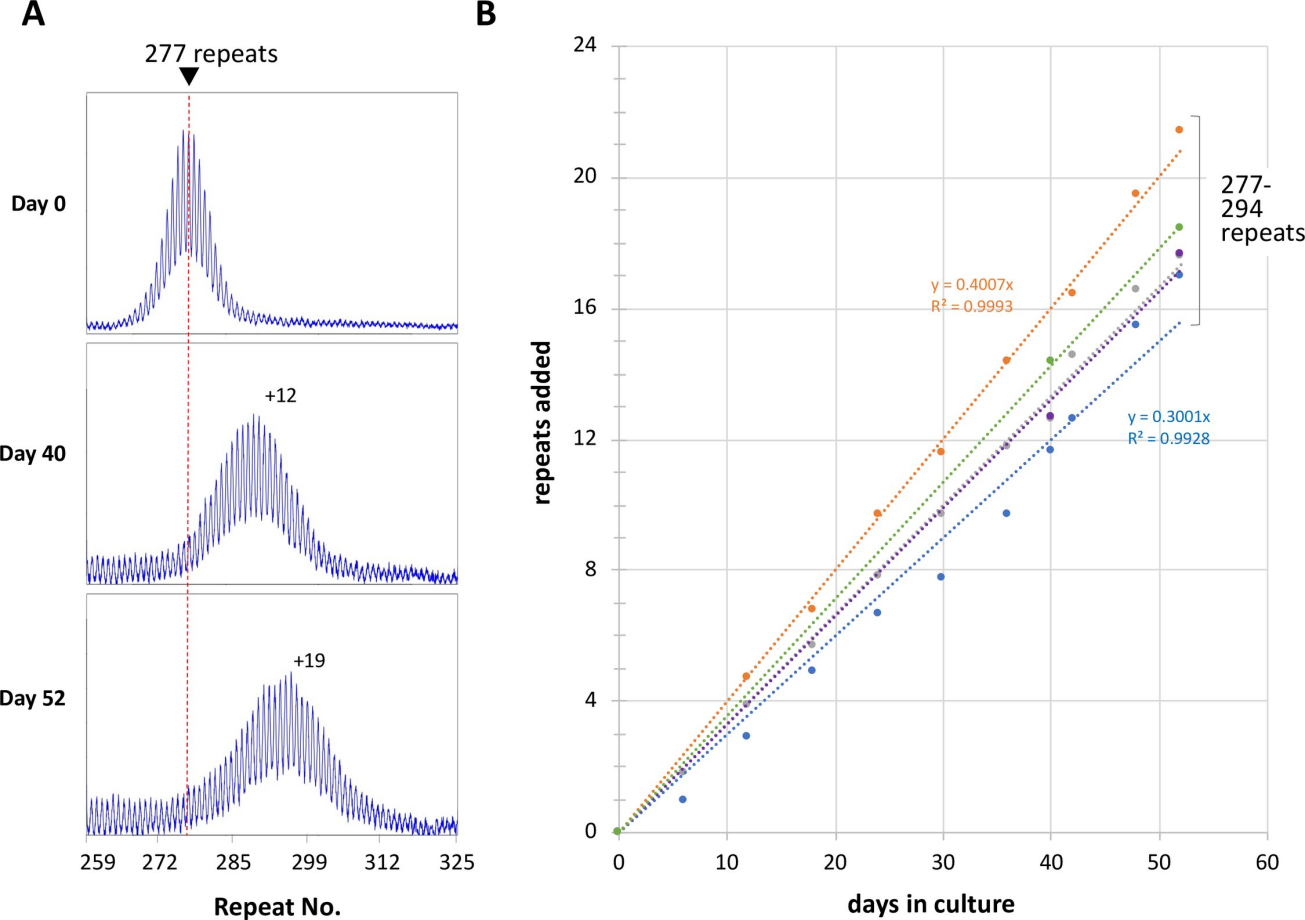
Repeat expansion in mESCs with ~280 repeats during time in culture. A) PCR profiles of a cell line WT for *Mlh3*, *Pms2* and *Pms1* with a starting allele of 277 repeats after 0, 40 and 52 days in culture. The red dotted line indicates the major allele present at day 0. B) Repeats added as a function of time for the cell line shown in panel A together with four additional lines with initial repeat numbers of 286–294. Only the trendline equation and R^2^ values for the fastest and slowest expanding lines are shown.

We have previously shown that mESCs from FXD mice show a progressive, length-dependent increase in the number of CGG-repeats in the *Fmr1* allele with time in culture [33]. To maximize the sensitivity of our assay and thus the ability to reliably detect even relatively small changes in the expansion frequency, we focused on mESC lines with ~280 repeats. Unlike similarly sized alleles in humans, these alleles do not become methylated, and as a result they continue to expand (Fig 1A). As with cell models of other REDs [24, 34, 35] and in expansion-prone cells of human and mouse PM carriers [36], all cells in the population expand more or less in concert. This reflects the high expansion frequency coupled with the fact that most alleles gain only one or two repeats with each expansion event [37]. Thus, the gain of ~19 repeats after 52 days in culture seen for the cell line shown in Fig 1A demonstrates not only that most alleles in the population have expanded, but also that those alleles in the population that have expanded have done so between ~10 and 19 times, amounting to many hundreds of thousands of independent mutational events.

To assess any cell line variation that there may be in the expansion rate we evaluated repeat expansion in four additional independently derived mESC lines with initial repeat numbers ranging from 287–294 repeats (Fig 1B, Table 1). A frequent mutational event operating stochastically on different alleles in the population results in an increase in the heterogeneity of allele sizes over time; and an accurate determination of the average repeat number becomes increasingly difficult, as is beginning to be apparent in the last panel of Fig 1A. We thus limited the analysis to cell lines grown for 52 days or less. We found that the wild-type lines added an average of 18±1.7 repeats in 52 days (Table 1). That corresponds to the addition of about one repeat every 2.8 days. The Expansion Index, a sensitive measure of the extent of expansion, was calculated as described in the Materials and Methods section. This metric was 1 or below at day 0, consistent with the fact that these were all early passage cells and by day 52 the cell lines had an average Expansion Index of 14.3 (Table 1).

**Table 1:** Instability characteristics of WT, *Mlh3*^-/-^, *Pms*1^-/-^ and *Pms*2^-/-^ mESCs.

Genotype	Cell Line	Days	Repeat no.	Δ no.	EI*	Δ EI
WT	(ave)	0	287		0.3	
		52	305	18	14.3	14.0
*Mlh3*^-/-^	#1	0	280		-1.0	
		52	279	-1	-2.0	-1.0
	#2	0	282		-0.9	
		52	282	0	-1.2	-0.3
*Pms1*^-/-^	#1	0	274		-1.1	
		52	272	-2	-2.6	-1.5
	#2	0	279		-0.9	
		52	277	-2	-2.0	-1.1
*Pms1*^-/-^	#1	0	261		-0.3	
		52	261	0	0.2	0.5
	#2	0	275		-0.5	
		52	276	0	-0.1	0.4

*Expansion Index

To examine the effect of mutations in the MLH1 binding partners we used CRISPR-Cas9 to generate null lines from early passages of mESCs with ~280 repeats. Although we had previously demonstrated a requirement for MLH3 in repeat expansion in a variety of mouse tissues [13], we also generated *Mlh3* null lines to specifically assess the requirement of MLH3 in embryonic stem cells and to facilitate comparison with the effects of mutations in PMS2 and PMS1. The derivation of the *Mlh3*, *Pms2* and *Pms1* null lines is described in detail in the Methods section. For each of the three CRISPR-Cas9-targeted genes, we selected two independent cell lines for further study, each having biallelic null mutations of the targeted gene and repeat numbers similar to the WT cell lines described above.

**Fig 2:**
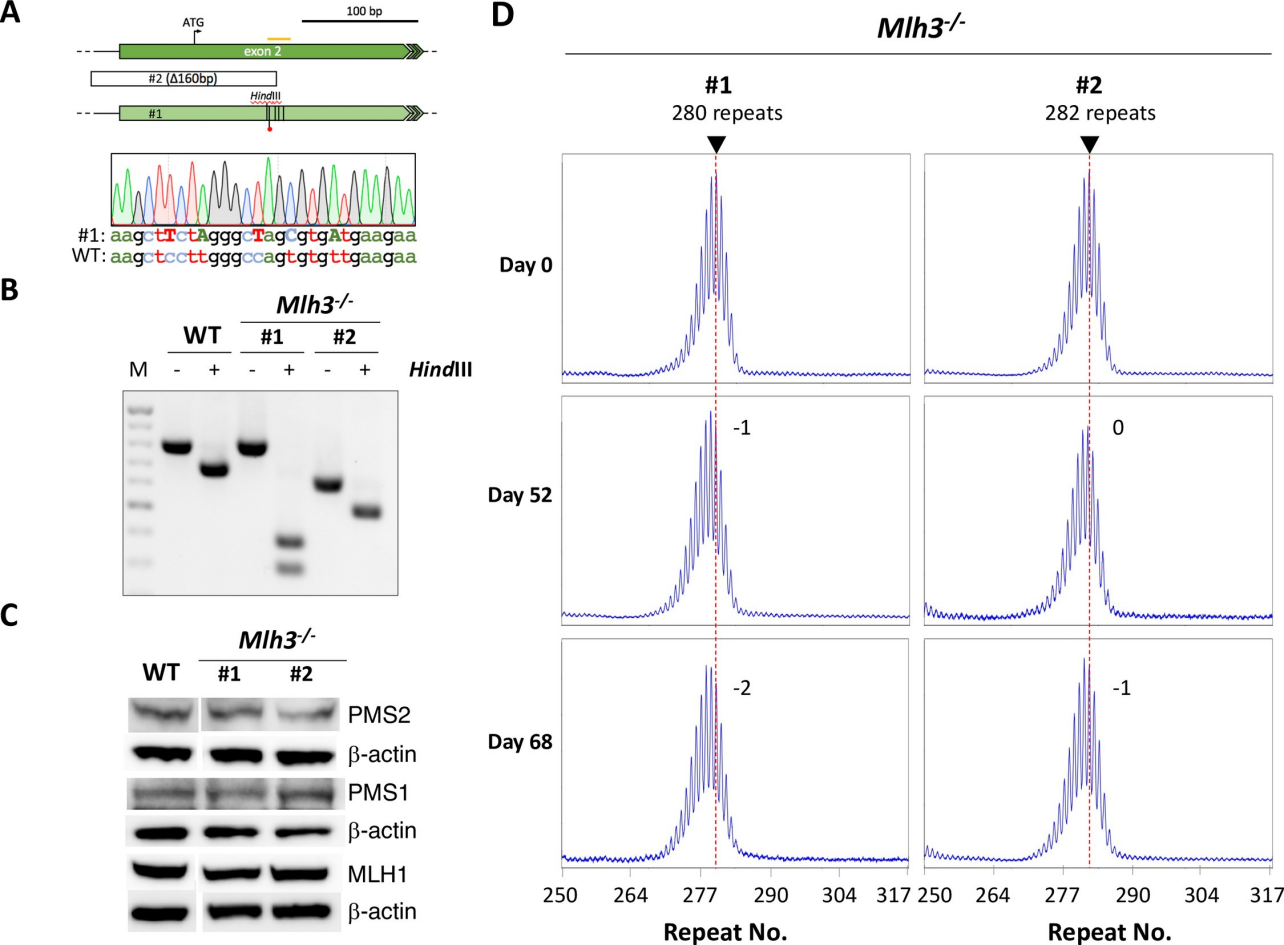
Generation and analysis of *Mlh3*^-/-^ cell lines. A) Diagram of mutations generated in exon 2 (the first coding exon) of the *Mlh3* locus. Orange bar: CRISPR gRNA target. The ATG indicates the initiator methionine. Light-shaded arrow: HDR-edited exon in *Mlh3*^-/-^ #1 mutant line. Vertical lines within the arrow indicate the position of HDR generated point mutations. The red dot indicates the stop codon introduced by point mutations. The red squiggle marks the position of the HDR ssODN. Open box: extent of CRISPR-induced deletion in #2 line. The sequencing trace shows the sequence of this region in line #1 with the bases shown in capitals being the edited bases. B) PCR and restriction digestion of DNA from *Mlh3*^+/+^ cells and two *Mlh3*^-/-^ CRISPR-edited cell lines. *Hin*dIII cuts the PCR products from WT and mutant lines to produce a 95 bp fragment (not shown). It also makes a second cut in the PCR product from the HDR-edited allele of line #1, but not the deleted allele of line #2. M: 100 bp molecular weight ladder. C) Western blot demonstrates that the loss of MLH3 does not affect the levels of MLH1, PMS1 or PMS2. Note that the lanes shown here are from the same blots shown in Figs 4C and 5C. D) Repeat PCR profiles of the two *Mlh3*^-/-^ lines after 0, 52 and 68 days in culture. The red dotted line on each profile indicates the major allele present in the cell population at day 0. The numbers adjacent to the profiles indicate the change in the repeat number relative to day 0.

To generate the *Mlh3*^-/-^ lines we employed a homology-directed repair (HDR) strategy. As can be seen in Fig 2A and 2B, one of the *Mlh3*^-/-^ lines (#1) was homozygous for both a novel *Hin*dIII site and an in-frame stop codon consistent with successful HDR in the highly conserved first coding exon whilst the second line (#2) carries a large deletion. The original repeat numbers were 280 and 282 respectively. The lack of suitable antibodies for detecting mouse MLH3 has been reported previously [38] and our own testing of multiple MLH3 antibodies also did not identify any suitable for verifying the *Mlh3*^-/-^ lines by western blot. Loss of MLH3 had no effect on the levels of MLH1, PMS1 or PMS2 (Fig 2C), consistent with previous reports and with the idea that loss of MLH3 does not affect the levels of MutLα and MutLβ [39].

In contrast to the expansions seen with WT cells, no evidence of expansion was seen in the *Mlh3*^-/-^ cell lines after 52 days in culture (Fig 2D), consistent with our previous demonstration that MLH3 is required for expansion in all mouse tissues [13]. Rather than the large increase in the Expansion Index seen in WT cells, the *Mlh3*^-/-^ cells instead showed a very small decline (-1.0 and -0.3 respectively, Table 1) suggestive of a subtle shift in the population of alleles toward smaller repeat numbers. Since the repeat does not expand, the repeat PCR profile remains quite homogeneous and it was thus possible to propagate the cells longer than 52 days. After 68 days in culture, the average repeat size actually decreased very slightly as suggested by the Expansion Index at 52 days.

**Fig 3:**
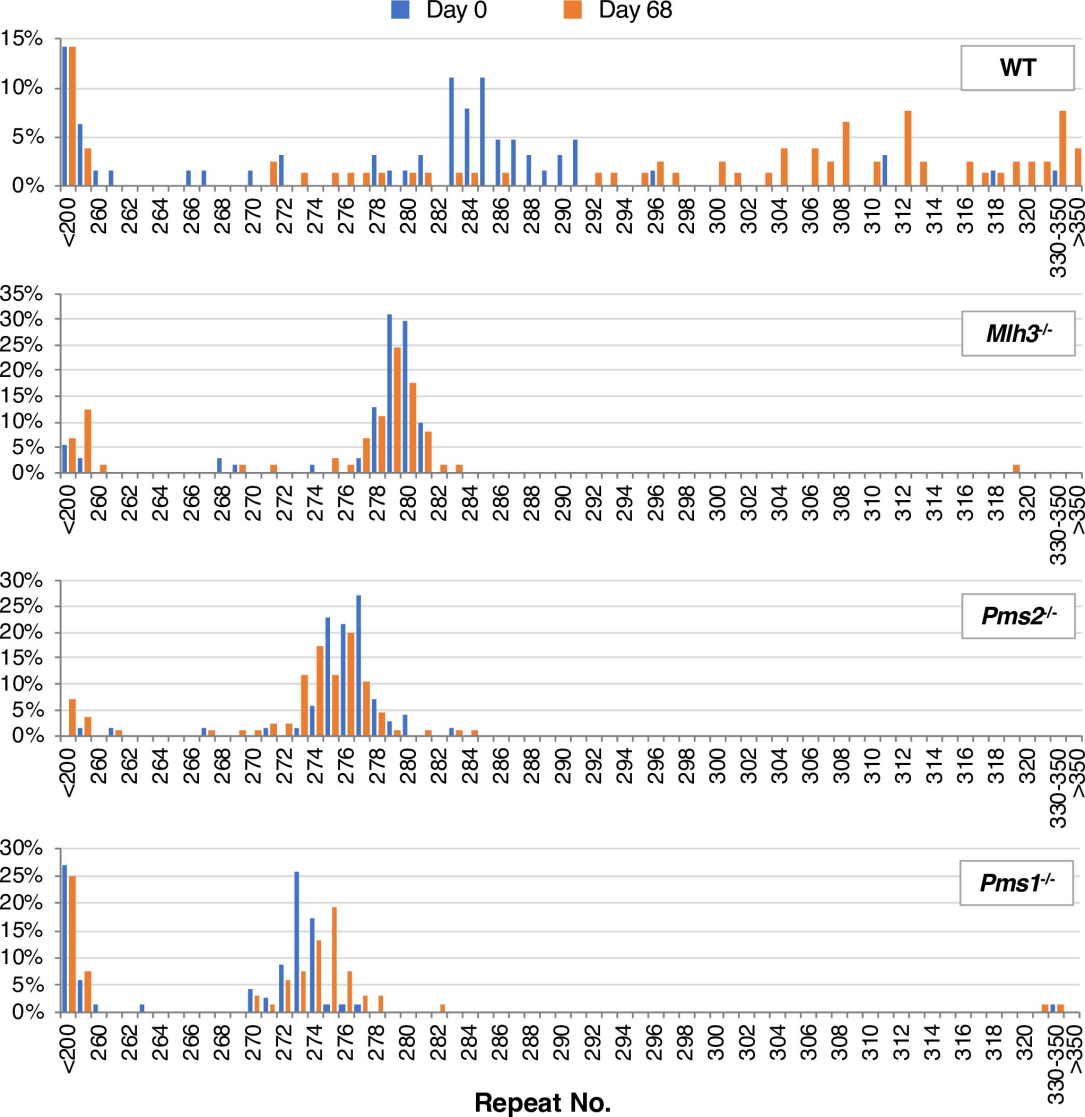
Small Pool PCR analysis of DNA isolated from WT, *Mlh3*^*-/-*^, *Pms2*^*-/-*^ and *Pms1*^*-/-*^ lines at day 0 and day 68. The day 0 data is shown with the blue bars, the day 68 data with orange bars. One line was used for each genotype (#2 for WT, *Mlh3*^-/-^ and *Pms1*^*-/-*^ and #1 for *Pms2*^*-/-*^) and 68–86 alleles were assessed for each line. Note that the nested PCR required for SP-PCR results in a left shift of the modal repeat number by two repeats relative to the number seen in the bulk PCR, likely because of additional strand-slippage during the large number of rounds of PCR involved.

Large expansions and contractions are typically underrepresented in standard PCR. We thus also used small pool PCR (SP-PCR) to compare the allele distributions at day 0 and day 68 in WT and *Mlh3*^-/-^ lines. SP-PCR has been used effectively to examine repeat expansion in other REDs [30]. It uses template DNA at limiting dilution and thus can detect individual alleles that do not fall into common size classes. As can be seen in Fig 3, WT cells had a significantly different allele distribution at the two time points (p<0.001), with virtually no trace of the original allele visible at day 68. The *Mlh3*^-/-^ SP-PCR data was strikingly different. Firstly, no difference was seen in the overall allele distributions of the *Mlh3*^-/-^ lines at day 0 and day 68 (Mann-Whitney’s U test; p = 0.10; Fig 3) and just a single unequivocally expanded allele was seen at day 68. This suggests that most expansions are dependent on the presence of MLH3, with the single expanded allele perhaps reflecting a limited ability of PMS2 or PMS1 to compensate for the loss of MLH3 in the expansion process.

**Fig 4:**
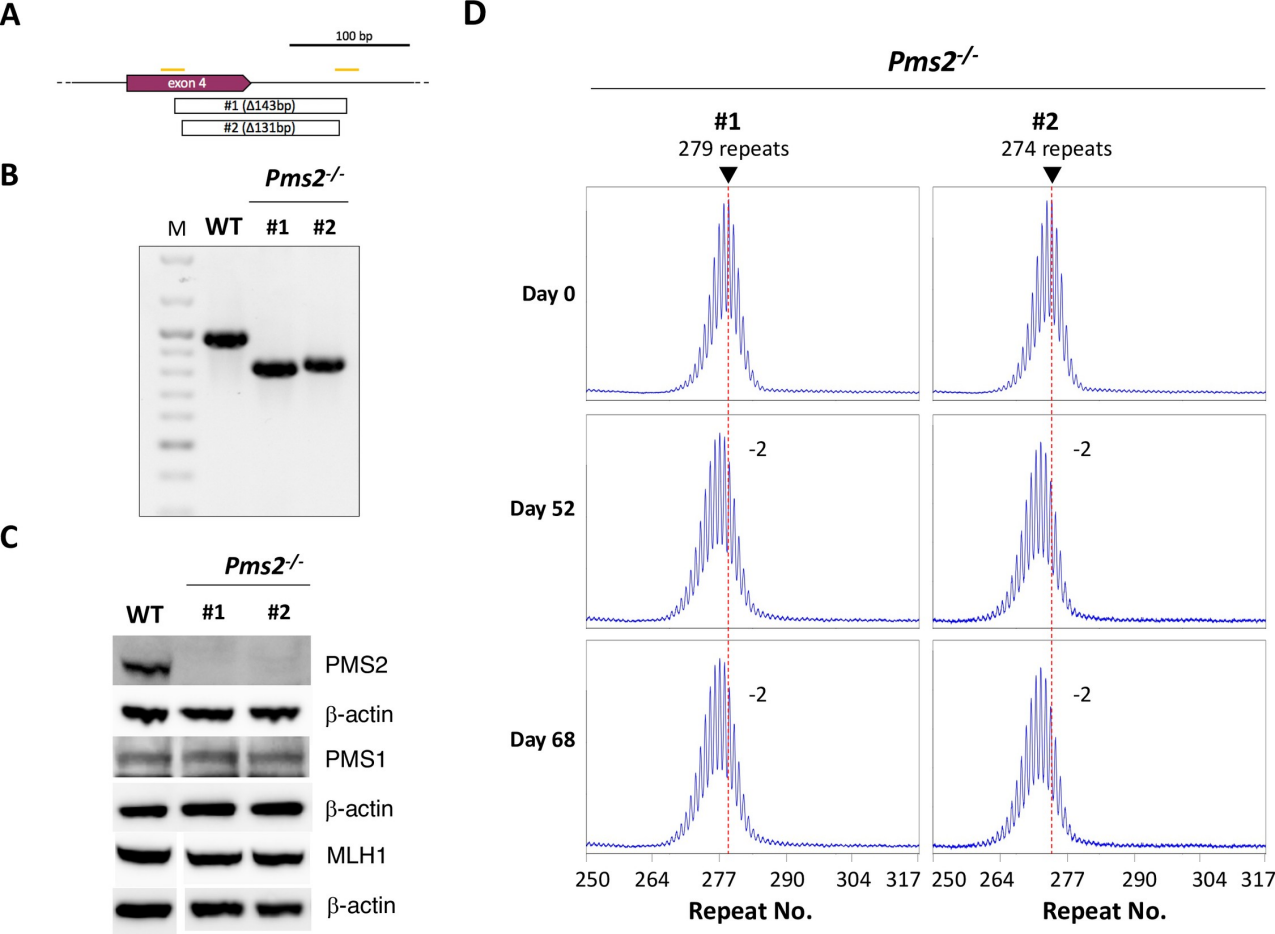
Generation and analysis of *Pms2*^-/-^ cell lines. A) Diagram of mutations generated in exon 4 of *Pms2*. Orange bars: CRISPR gRNA target. Open boxes: extent of CRISPR-induced deletion in the indicated lines. B) PCR analysis of DNA from *Pms2*^+/+^ cells and the two *Pms2*^-/-^ CRISPant cell lines shown in panel A. M: 100 bp molecular weight ladder. C) Western blot demonstrating that the edited cells lack PMS2 and that the loss of PMS2 does not affect the levels of MLH1 or PMS1. Note that the lanes in the MLH1 western blot shown here are from the same blot shown in Figs 2C and 5C. D) Repeat PCR profiles of the two *Pms2*^-/-^ lines after 0, 52 and 68 days in culture. The red dotted line on each profile indicates the major allele present in the cell population at day 0. The numbers indicate the change in the repeat number relative to day 0.

In parallel with the *Mlh3*^-/-^ lines, we examined expansion in two *Pms2*^*-/-*^ mESC lines, one with 279 repeats and another with 274 repeats. As can be seen in Fig 4A and 4B, cell lines #1 and #2 had a 143 bp and a 131 bp deletion in *Pms2*, respectively. Both deletions span the 3’ exon/intron boundary of exon 4. No residual PMS2 was detected in either cell line (Fig 4C). Furthermore, consistent with previous reports from other cell types [40, 41], no change in the levels of MLH1 or PMS1 was seen in either cell line (Fig 4C). Thus, these mutations are unlikely to negatively affect the levels of either PMS1 or MLH3.

Examination of the repeat PCR profiles at day 0 and day 68 showed that neither of the *Pms2*^*-/-*^ cell lines had gained any repeats over this period (Fig 4D). In fact, a loss of two repeats was apparent by 52 days and the Expansion Index shows a similar negative change (-1.5 and -1.1 respectively; Table 1). As shown in Fig 3, SP-PCR of DNA at day 0 produces a very similar distribution of alleles as was seen for the *Mlh3*^-/-^ line. At day 68 there was a small increase in the number of alleles that had lost a few repeats consistent with what was seen using bulk PCR. Just one allele was seen at day 68 that was one repeat larger than the largest allele seen at day 0, but whether it really represents an expansion is unclear. The elimination of most, if not all, expansions indicates that PMS2, like MLH3, is important for expansions in the FXD mESCs. The loss of a few repeats could be consistent with the role of MutLα in MMR protecting against MSI, with the loss of PMS2, like the loss of MLH3, resulting in a mutational bias towards contractions at this CGG-microsatellite.

**Fig 5:**
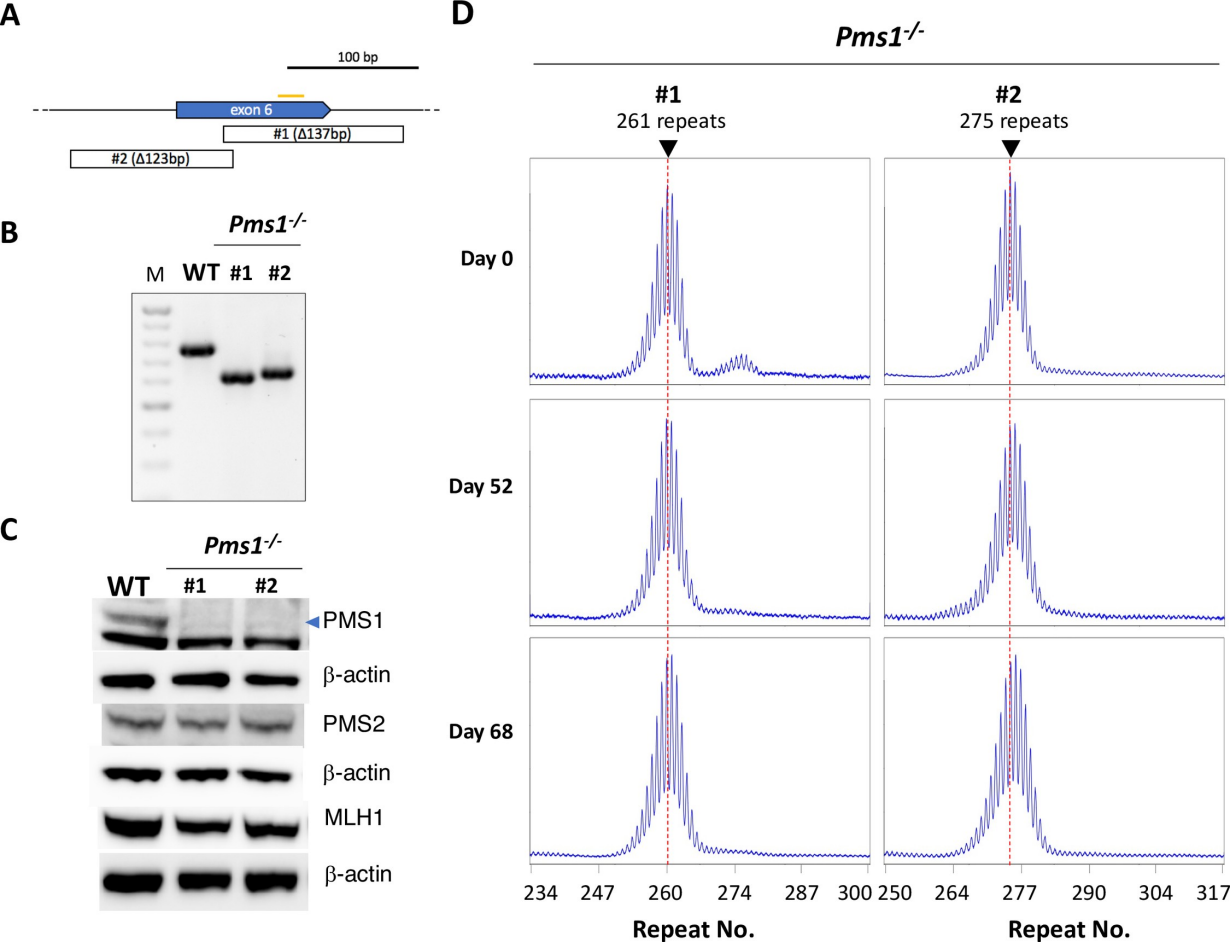
Generation and analysis of *Pms1*^-/-^ cell lines. A) Diagram of mutations generated in exon 6 of *Pms1*. Orange bar: CRISPR gRNA target. Open boxes: extent of CRISPR-induced deletion in the indicated lines. B) PCR analysis of DNA from *Pms1*^+/+^ cells and the two *Pms1*^-/-^ CRISPant cell lines shown in panel A. M: 100 bp molecular weight ladder. C) Western blot showing the deficit of PMS1 in the edited lines and that the loss of PMS1 does not affect the levels of MLH1 or PMS2. Note a) that the faint band apparent in the *Pms1*^*-/-*^ lanes of the PMS1 blot corresponds to the smaller of the two closely migrating bands seen in *Pms1*^+/+^ lines and b) that the lanes from the MLH1 western blot shown here are from the same blot shown in Figs 2C and 4C. D) Repeat PCR profiles of the two *Pms1* mutant lines after 0, 52 and 68 days in culture. The red dotted line on each profile indicates the major allele present in the cell population at day 0.

For analysis of the effect of PMS1 loss on repeat expansion we used one cell line with 261 repeats (#1) and a second with 275 repeats (#2). Cell line #1 had a 137 bp deletion in *Pms1* that removed most of exon 5 including the 3’ exon-intron boundary and had an in-frame stop codon, while cell line #2 had a 123 bp deletion that removed the 5’ end of the exon (Fig 5A and 5B). Both cell lines showed depletion of PMS1 and had levels of MLH1 and PMS2 that were indistinguishable from WT cells consistent with previous reports (Fig 5C) [39].

As was seen with the *Mlh3*^-/-^ mutant lines, there was no significant change in the repeat profile at 52 days in either *Pms1*^*-/-*^ cell line. There was also no change in the Expansion Index for either *Pms1*^*-/-*^ cell line. At day 68 there was a slight shift in the PCR profile (Fig 5D) and a small change in the Expansion Index consistent with the addition of one to two repeats to a fraction of cells in the population (Table 1). SP-PCR also shows a small shift in the distribution of alleles at day 68 that is consistent with the gain of a one or two repeats (Fig 3). However, again it is unclear whether these small increases are the result of *bona fide* expansion events. It is possible that they reflect a MMR-related role for PMS1 in preventing MSI as previously suggested [42], with the loss of PMS1 showing an insertion bias at this microsatellite. Whatever the molecular basis of these small repeat additions, the fact that it takes 68 days for these alleles to become apparent suggests that they are occurring at a much lower frequency than the expansions seen in the WT lines. At day 68 there were also a few more alleles with large expansions than were seen at day 0. As suggested for the expanded allele seen in SP-PCR of *Mlh3*^-/-^ cells, these residual larger alleles may reflect the ability of one of the other MutL proteins to compensate, albeit relatively poorly, for the loss of MutLβ. A number of alleles with large contractions were also seen. However, a similar number of such alleles were seen at day 0 and their significance is unclear. In any event, it is apparent that, as with the MLH3 and PMS2 mutant lines, most alleles that have not contracted have not expanded either, and that PMS1, like MLH3 and PMS2, plays an important role in repeat expansion.

## Discussion

Here we show that all three known MLH1-binding partners, MLH3, PMS2 and PMS1, are required for the vast majority of repeat expansions in a mouse ESC model of the FXDs. The small number of residual expansions seen in the individual mutant lines may reflect the ability of one or more of these proteins to substitute, albeit inefficiently, for one another. A requirement for all three proteins is interesting given that human GWA studies have also implicated all of these proteins as genetic modifiers of expansion risk in other REDs [5, 7, 9, 10, 29, 43]. This suggests that the FXD mESC model system might properly model the mutation responsible for the human REDs. The importance of MLH3 and PMS1 is all the more remarkable given that PMS2 has been estimated to be present at levels ~10 times higher than PMS1 and 60–400 times higher than MLH3 [26–28]. In fact, while PMS2 is relatively abundant, MLH3 has been estimated to be present in as few as ~110 molecules per cell [27]. We have previously shown that even heterozygosity for *Mlh3* is rate limiting in a mouse model [13]. While it is reasonable to think that the same would be true in mESCs derived from these mice, it would not necessarily be the case for *Pms1* or *Pms2* since they are so much more abundant than MLH3.

The requirement for all three proteins would be consistent with a model in which each of the MutL complexes have their own unique functions that are essential for expansion. For MutLγ, this could be its affinity for the expansion substrate [44] or the nature of the cleavage products it generates [45]. Since the MutLα nuclease generates cleavage products that differ from that of MutLγ [45, 46], it is possible that its nuclease activity is also required. While MutLβ has no identified nuclease motifs, it is possible that MutLβ, and perhaps also MutLα, have specific structural roles in expansion, analogous perhaps to the roles they play alongside MutLγ in meiosis [47, 48]. However, if all REDs do indeed share a common mechanism, a model in which all three complexes make unique contributions to the expansion process is inconsistent with the observation that the loss of MutLα does not cause a loss of expansions in some model systems [24]. Since MLH3 is by far the least abundant of the MLH1-binding partners and the MLH3 nuclease is required for expansion [49], we favor a model in which MutLγ plays a unique role and provides an essential catalytic activity in the expansion process. In contrast, we suggest that MutLα, and perhaps MutLβ, are only able to carry out a subset of the MutLγ activities required for expansion. For example, we know that in the case of yeast MutLγ, polymer formation is required for MutLγ cleavage [50]. Polymer formation is a conserved property of MutL complexes in organisms ranging from bacteria to mammals, including human MutLα and yeast and human MutLγ [50, 51]. Given the sequence similarities and many common properties of the MutL proteins, we speculate that MutLα and MutLβ have some ability to contribute to the polymers formed on a MutLγ-bound substrate. In this scenario, an effect of the loss of MutLα and MutLβ would only be seen when MutLγ was rate-limiting, for example in the presence of a large amount of the expansion substrate. Loss of MutLα in cases where MutLγ was not rate-limiting could even result in increased repeat instability by virtue of the loss of the protection against MSI that MutLα normally provides. This model makes a number of interesting predictions that can now be tested.

It remains to be seen whether MutLβ is required for expansions in all REDs, although GWA studies suggest it might be important for a large subset at least. A better understanding of the role of MutLβ in the context of these expansions may help shed light on its role in expansion as well as its normal, and currently somewhat enigmatic, cellular role. In addition, while mutations in MSH3 and MLH3 have been implicated in a variety of cancers and cancer predisposition syndromes [52–57], PMS1 mutations have not been definitively associated with any cancer predisposition to date either in mice [42] or humans (see omim.org/entry/600258?search=pms1&highlight=pms1). Thus, should our findings in the FXD mouse model translate to humans, PMS1 may be a reasonable therapeutic target in those diseases where somatic expansion is an important disease modifier.

## Materials and methods

### Reagents and services

pSpCas9(BB)-2A-Puro (PX459) V2.0 was a gift from Feng Zhang (Addgene plasmid # 62988; http://n2t.net/addgene:62988; RRID:Addgene_62988) [58]. Standard reagents were from Sigma-Aldrich unless otherwise specified. Cell culture reagents were from ThermoFisher Scientific except as noted. Short double stranded oligonucleotides used for cloning the guide RNAs as well as sequencing and PCR primers were purchased from ThermoFisher Scientific. Single-stranded oligonucleotides (ssODNs) and the scaffold-U6prom dsDNA fragment were synthesized by Integrated DNA Technologies. All the oligonucleotides used to generate the mutant mESCs are listed in Table 2. DNA sequencing and capillary electrophoresis of fluorescently labeled PCR products was carried out by Psomagen.

**Table 2:** Oligonucleotides used to generate the *Mlh3*^-/-^, *Pms*1^-/-^ and *Pms*2^-/-^ mESCs.

Name	Oligo sequence (5' to 3')
Mlh3_gRNA-F	CACCGTTCTTCAACACACTGGCCCA
Mlh3_gRNA-R	AAACTGGGCCAGTGTGTTGAAGAA
Mlh3_ssODN	TCTATCAGATGACGTAAAAACCAAGTTGCGTTCCGGTTTAGCCATAAGCT**T**CT**A**GGGC**T**AG**C**GTG**A**TGAAGAACTTACCCTTAACAGTATTGATGCTGAAGCAACATGTGTGGCCA
Pms1_gRNA-F	CACCGCAGAGTTCCAGATCACAGGA
Pms1_gRNA-R	AAACTCCTGTGATCTGGAACTCTG
Pms1_ssODN	TTCCATGTTGCCCATGACAGCAGTTCCCAGAACCGACATTAGAG**GG**ATCCT**AA**GATC**A**GGAAC**A**CTGCTTT**A**CTGCCAAATAACTGCCTAGGGAGAAGAAAACATAGATGTGTTGTAGCACA
Pms2-gRNA1-gibson	GAAAGGACGAAACACCGAGAGTTTGCCGACCTCACGCGTTTTAGAGCTAGAAATAGC
Pms2-gRNA2-gibson	TTTCTAGCTCTAAAACCGTGGTTTATGCTTTACTACGGTGTTTCGTCCTTT
Pms2-ssODN	CTTTCTACTCTCTTTCAGCTCTGAAACATCACACATCTAAGATTCAAGAGTTTGCCGTGCTTTACTACTGAAGTATCTTGACTCCGAGCTTTAATTCTCT
scaffold-U6prom	GTTTTAGAGCTAGAAATAGCAAGTTAAAATAAGGCTAGTCCGTTATCAACTTGAAAAAGTGGCACCGAGTCGGTGCTTTTTTTCCGATCATGGGTCGAACGTTACGCAGAGGGCCTATTTCCCATGATTCCTTCATATTTGCATATACGATACAAGGCTGTTAGAGAGATAATTGGAATTAATTTGACTGTAAACACAAAGATATTAGTACAAAATACGTGACGTAGAAAGTAATAATTTCTTGGGTAGTTTGCAGTTTTAAAATTATGTTTTAAAATGGACTATCATATGCTTACCGTAACTTGAAAGTATTTCGATTTCTTGGCTTTATATATCTTGTGGAAAGGACGAAACACCG

Note: the underlined bases indicate the gRNA sequences and the bases in bold indicate the mutations introduced in the ssODNs to generate stop codons in all 3 reading frames.

### Generation of *Mlh3*^-/-^, *Pms1*^-/-^ and *Pms2*^-/-^ mESCs

Null mutations in *Mlh3*, *Pms2*, and *Pms1* were generated in established mESC lines [33], using CRISPR-*Cas9* and either a single or double gRNA strategy. All gRNAs chosen had a low risk of significant off-target editing as assessed using a variety of algorithms including E-CRISP (http://www.e-crisp.org/E-CRISP/reannotate_crispr.html; [59]), with the only potential hits elsewhere in the genome having multiple sequence mismatches. None of those potential off-target hits were in genes involved in DNA replication, repair or recombination (see S1 Data). Thus, even in the unlikely event that off-target editing occurred at significant frequencies in our cell lines [60–62], they are unlikely to account for any effects observed in the mutant cells used here. Nonetheless, two independently derived gene-edited cell lines were used for each mutation to reduce this possibility even further.

Specifically, the *Mlh3* and *Pms1* mutated cell lines were generated using a single gRNA strategy with the GUIDES algorithm being used to identify suitable gRNAs [63]. Oligonucleotide pairs were designed that specified the gRNAs and that would generate 5’ overhangs compatible with *Bbs*I digested PX459 v2.0 [58]. These oligonucleotide pairs, Mlh3_gRNA-F/Mlh3_gRNA-R and Pms1_gRNA-F/Pms1_gRNA-R respectively (Table 2), were annealed and cloned into *Bbs*I digested PX459 v2.0 [58]. To generate *Pms2* mutant lines, Benchling (www.benchling.com) was used to identify two different gRNAs. The primers Pms2-gRNA1-gibson and Pms2-gRNA2-gibson, containing the gRNA-specifying sequences, were used to PCR amplify from the scaffold-U6prom template (Table 2) which contains a gRNA scaffold and U6 promoter. The resulting PCR product was then cloned using Gibson Assembly into *Bbs*I digested PX459 v2.0 [58] resulting in a plasmid expressing the two gRNAs each with their own gRNA scaffold and promoter. All gRNA-encoding plasmids were verified by DNA sequencing.

These plasmids were used together with ssODNs designed so as to facilitate the introduction of in-frame stop codons via homology directed repair (HDR; *Pms1* and *Mlh3* target sites) or deletion of the region between gRNAs (*Pms2* target site) (Table 2). The DNAs were electroporated into ~2x10^6^ mESCs using the AMAXA electroporator and the Mouse Embryonic Stem Cell Nucleofector kit (VAPH-1001, Lonza) as per the manufacturer’s recommendations. Puromycin at 1μg/ml was added 24 hours after electroporation and increased to 2 μg/ml 24 hours after that. After an additional 24 hours, the medium was replaced with puromycin-free medium. Cell lines were established from individual isolated colonies, and from these, successfully edited cell lines were identified by direct sequencing of the PCR product. In all the lines chosen a homogeneous sequence profile was seen that was consistent with either a biallelic mutation or a second mutation that deleted one or both of the PCR primer binding sites. The only detectable allele in each case contained a deletion that changed the reading frame and/or deleted an exon/intron boundary or had an HDR-mediated point mutation that inserted an in-frame stop codon. Edited cell lines and WT control lines with the similar repeat numbers were chosen for further analysis.

### Western blotting

Cells were resuspended in RIPA lysis buffer (150 mM NaCl, 1.0% NP-40, 0.5% deoxycholate, 0.1% sodium dodecyl sulfate, 50 mM Tris pH 8.0) supplemented with 1X protease inhibitor cocktail (P8340, Sigma-Aldrich). Cells were sonicated for 5 times for 10 sec each with a 10 sec rest using a Branson Sonifier 250 at 40% power. Lysates were then centrifuged for 5 min at 10,000xg at 4°C. The protein concentration in the supernatant was determined using Bio-Rad Protein Assay Dye Reagent Concentrate (5000006, Bio-Rad). Between 20–30 μg of protein were resolved on NuPAGE 3–8% (w/v) Tris-Acetate gels (ThermoFisher Scientific) and transferred onto nitrocellulose membrane using a wet-transfer apparatus using a buffer containing 10% methanol and 90% 1x transfer buffer (NP00061, ThermoFisher Scientific) at 0.4A for 90 min in a cold room. The membrane was blocked with 5% ECL Prime Blocking Agent (RPN418, GE Healthcare) in TBS-T (20 mM Tris-HCl (pH 7.4), 150 mM NaCl, 0.1% (v/v) Tween-20). The sources and dilutions used for the antibodies to MLH1, PMS2, PMS1, β-actin and the horseradish peroxidase–conjugated secondary antibody are listed in Table 3. Membranes were incubated with the antibodies overnight at 4°C, then washed 5 times in TBS-T before incubation with horseradish peroxidase–conjugated secondary antibody. For detection either the ECL Plus chemiluminescence reagent (RPN2232, GE Healthcare) or SuperSignal West Femto Maximum Sensitivity Substrate (34094, ThermoFisher Scientific) was used. The blots were imaged using a ChemiDoc imaging system (Bio-Rad).

**Table 3:** Antibodies used.

Antigen	Source	Catalog No.	Dilution
MLH1	Abcam	ab92312	1:2,000
PMS2	Santa Cruz Biotechnology	sc618	1:200
PMS1	MyBioSource	mbs9134056	1:500
β-actin	Abcam	ab8227	1:10,000
ECL Rabbit IgG, HRP linked	GE Healthcare	NA934	1:2,500

### Analysis of repeat expansion

Cell lines were grown in N2B27 2i/LIF medium with passaging every other day onto gelatin-treated tissue culture plasticware as previously described [33]. DNA samples were taken at regular intervals for analysis of the repeat PCR profile. This profile was determined as previously described using a PCR assay and high-resolution capillary electrophoresis [13] and compared to the original repeat number present at Day 0. The Expansion Index, a variation of the Somatic Instability Index [64] that only considers data from the highest peak to the peak corresponding to largest allele that is still above the threshold, was calculated for each time point. This index is a very sensitive measure of the extent of expansion and can detect even very small changes in the average repeat number in the population. Small Pool PCR (SP-PCR) was carried out using a nested PCR strategy as previously described [65].

## Supporting information

S1 Data Predicted off-target sites of gRNAs used in this study. Excel workbook containing lists of the most likely gRNA off-target hits for the gRNAs used to generate the *Pms1*, *Pms2* and *Mlh3* null cell lines.
(XLS)
